# Arthroplastie totale du coude sur séquelles d'ostéoarthrite négligée-à propos d'un cas

**DOI:** 10.4314/pamj.v9i1.71220

**Published:** 2011-08-22

**Authors:** Abdelhalim El Ibrahimi, Mohammed Elidrissi, Mohammed Shimi, Abdelmajid Elmrini

**Affiliations:** 1 Service de Chirurgie Ostéoarticulaire B4, CHU Hassan II de Fès, Fès, 30000 Maroc

**Keywords:** Arthroplastie, coude, ostéoarthrite, Maroc

## Abstract

L'arthroplastie totale au cours des séquelles d'infection ostéo-articulaire a été destinée surtout pour la hanche et le genou. Cependant, peu de travaux ont étaient consacrée pour l'articulation du coude. Nous rapportons le cas d'une patiente, âgée de 26 ans, ayant présentée dans l'enfance une ostéoarthrite du coude traitée traditionnellement, mais qui a développé progressivement une instabilité sévère du coude. Après l'implantation d'une prothèse semi-contrainte de Coonrad-Morrey non cimentée, le résultat est très satisfaisant jusqu’à deux ans de recul, avec un gain de la mobilité en flexion de 110 degrés et en extension de 5 degrés, l'index de performance pour le coude selon la Mayo Clinic de 80/100. Nous n'avons pas de complication à déplorer.

## Introduction

L'infection ostéo-articulaire du coude est un événement sérieux et grave responsable de remaniements articulaires entraînant la douleur et une altération fonctionnelle du coude. L'arthroplastie totale au cours des séquelles d'infection ostéo-articulaire a été destinée surtout pour la hanche et le genou [1–6]. Cependant, peu de travaux ont étaient consacrée pour l'articulation du coude. A travers une observation et une revue de la littérature, nous rapportons les résultats cliniques et radiologiques après implantation d'une prothèse totale semi contrainte du coude sur séquelles d'ostéoarthrite avec instabilité sévère.

## Cas Clinique

**Figure 1:**
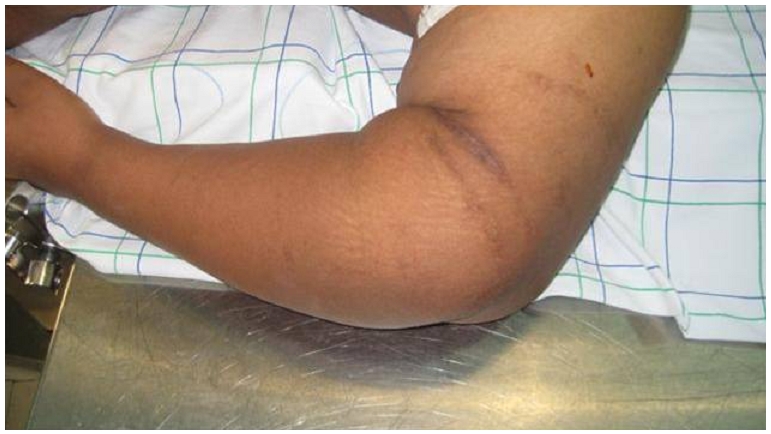
Aspect clinique préopératoire du coude avec les cicatrices de feu et des fistules

Une patiente âgée de 24 ans, droitière, sans profession consultait pour des douleurs du coude gauche avec instabilité sévère évoluant depuis l’âge de 8 ans. Elle était victime d'un traumatisme du coude n'ayant pas fait l'objet d'une prise en charge médicale, traitée à l’époque traditionnellement par du feu, l’évolution a été marquée par une ostéoarthrite du coude drainée et mise sous antibiotique. Au moment de la consultation, l'examen trouve à l'inspection des cicatrices de feu et de fistules en regard de la face postérieure du coude (Figure 1), le coude était presque ballant limitant considérablement les activités de la vie courante de notre patiente qui compensait en réalisant un grand nombre de gestes de la main droite.

**Figure 2:**
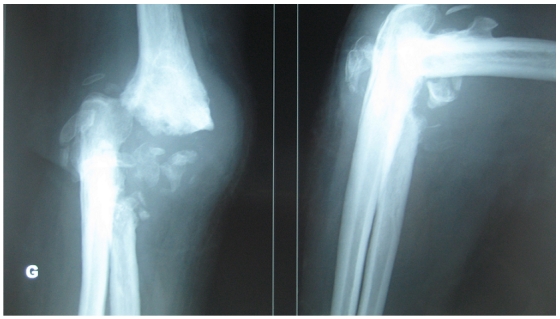
Radiographies de face et de profil du coude montant les séquelles post infectieuses

Le bilan radiographique objectivait un coude détruit avec une quasi-disparition de l'interligne articulaire et une déminéralisation osseuse diffuse (Figure 2). Un bilan infectieux complet comportant : CRP, VS et GB, est revenu sans particularités.

**Figure 3:**
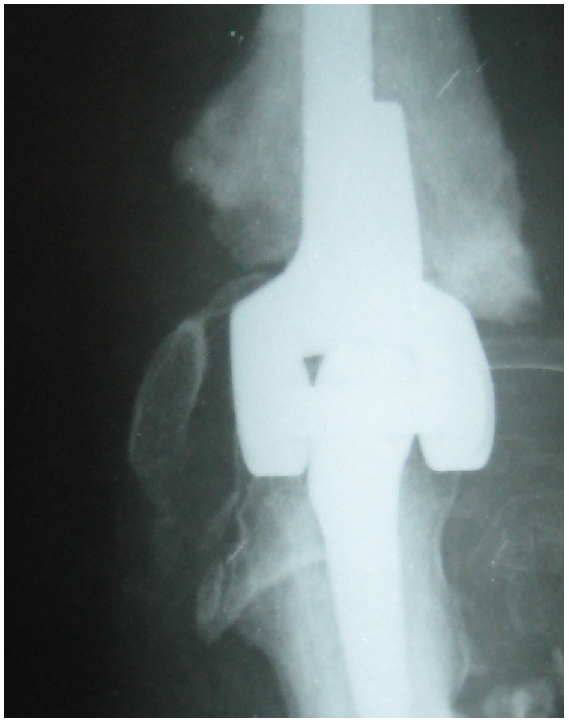
Radiographie postopératoire après implantation d'une prothèse totale du coude

Devant la demande fonctionnelle et les plaintes douloureuses de la patiente, malgré son jeune âge et l'ancienneté des séquelles, l'indication d'une arthroplastie semi-contrainte du coude de type Coonrad-Morrey était posée afin de récupérer l'indolence et une fonction correcte du membre. L'intervention était réalisée par voie d'abord postérieure longitudinale. Le nerf ulnaire a été neurolysé et transposé en avant de l’épitrochlée. Après résection de la tête radiale, préparation du fut huméral et cubital, Une prothèse Coonrad-Morrey de troisième génération (Zimmer) a été implantée. Il s'agit d'une prothèse totale semi-contrainte munie d'un aileron antérieur à l'extrémité distale afin prévenir la migration postéro-supérieure de l'implant. Les pièces humérale et ulnaire étaient mises en place sans ciment (Figure 3). En peropératoire, une mobilité complète fut obtenue en flexion et en extension. Enfin, une attelle plâtrée postérieure était mise en place pour trois semaines. Celle-ci maintenait le coude dans une position fléchie de 50°. La rééducation a été entreprise de façon biquotidienne au lendemain de l'intervention sous couverture d'une analgésie par cathéter péri nerveux et a été poursuivie après le retour à domicile.

## Discussion

Les séquelles d'infection ostéo-articulaire du coude sont responsables de douleur et de gène fonctionnelle. L’éventail thérapeutique fait appel à l'arthrodèse, la résection arthroplastie et à l'arthroplastie totale. Les Prothèses totales du coude ont été développées dans le cadre du traitement chirurgical de la polyarthrite rhumatoïde; mais dernièrement on voit leurs indications s’élargir mêmes aux séquelles post infectieuses. Si le remplacement prothétique a été étudié sur un milieu septique au niveau de la hanche et du genou, peu de travaux ont été consacrés à l'arthroplastie totale du coude chez des malades ayant une histoire infectieuse [1–6, 8–10].

De nombreuses études ont démontrées la possibilité et l'efficacité d'un remplacement prothétique de la hanche et du genou suite à une destruction articulaire secondaire à une infection ostéoarticulaire Le taux de réinfection est de 0 à 0,95% avec des résultats cliniques qui se rapprochent sans égaler ceux rencontrés sur une articulation sans histoire infectieuse. Dans ces séries, une évaluation préopératoire était nécessaire et faisait appel à la clinique, la biologie (GB, VS, CRP, Culture) et parfois à l'histologie. Cependant, des résultats histologiques positifs après arthroplastie ont conduits à maintenir la prothèse et à démarrer une antibiothérapie adaptée sous une étroite surveillance clinique et biologique [1–6].

Yamaguchi [7] rapporte dans une série de 10 patients traités par arthroplastie totale du coude semi contrainte sur coude infecté et documenté dont 7 cas sur coude prothèsé et uniquement deux cas sur séquelles d'ostéoarthrite du coude. L'intervalle entre le traitement de l'infection et l'implantation de la prothèse était de 45 mois (extrêmes 3 et 8 ans). Deux cas de récidive infectieuse ont été déplorés et concernent des coudes préalablement prothèsés. Les résultats fonctionnels étaient satisfaisants. Selon Yamaguchi [7], l'intervalle entre l'infection première et la date d'implantation de la prothèse n'est pas corrélé à la recrudescence de l'infection.

Serraa [11] a rapporté une observation d'un coude ballant post traumatique séquellaire d'une fracture-luxation datant de plus de 20 ans, avec des suites septiques pour laquelle une prothèse totale semi contrainte manchonnées par allogreffe massive a été implantée. A 75 mois de recul, le résultat clinique était excellent avec un index de performance de la Mayo Clinic de 100 points sur 100. Il n'existait aucune complication, notamment aucune récidive infectieuse ni descellement. Chez notre patiente l'infection ostéoarticulaire était non documentée, avec un intervalle de 17 ans entre l'infection et l'implantation de la prothèse. A deux ans de recul, aucun réveil infectieux ni descellement prothétique n'ont été noté.

Le contexte post-infectieux, habituellement à l'origine de résultats défavorables dans les séries d'arthroplastie de même qu'une forte demande fonctionnelle, ne doivent pas d'après nous faire contre-indiquer ce type de chirurgie si les impératifs anatomiques la rendent logique.

Pour mettre en place une arthroplastie totale du coude sur une articulation précédemment infectée, nous recommandons une évaluation préopératoire et peropératoire appropriée. Cette démarche vise à exclure une infection active et à déterminer les facteurs anatomiques qui peuvent influencer la chirurgie. Un bilan infectieux initial doit être effectué et toute anomalie devrait indiquée une étude scannographique. Cette dernière pourrait conduire à une aspiration ou à un débridement de l′articulation. En per-opératoire, des prélèvements doivent être effectués pour une étude cytobactériologique et histologique [1–4].

Nous rappelons toutefois que l'espérance de vie du patient ne met pas son coude à l'abri de complications tardives, infectieuses ou mécaniques. En outre, la technique utilisée constitue une solution de dernier recours et son indication chez des patients jeunes doit être mise en concurrence avec des solutions plus conservatrices [11].

## Conclusion

L'arthroplastie totale du coude sur séquelles d'ostéoarthrite représente un véritable challenge puisqu'elle est menée sur un milieu initialement infecté, un os de mauvaise qualité et des structures ligamentaires et musculaires déficientes. Cette attitude thérapeutique peut être réalisée avec un risque minimal de réinfection sous réserve d'une stricte sélection d'une articulation sèche et préalablement propre.
